# Formulation and Characterization of Hydrogel Chitosan–Pectin Active Films Containing Silymarin

**DOI:** 10.3390/molecules31020322

**Published:** 2026-01-17

**Authors:** Svetla Dyankova, Nadya Bozakova, Vanya Boneva, Ayten Solak, Veselin Ivanov

**Affiliations:** 1 Institute of Cryobiology and Food Technologies, Agricultural Academy, 1407 Sofia, Bulgaria; ayten.solak@ikht.bg; 2 Department of Animal Husbandry, Faculty of Veterinary Medicine, Trakia University, 6000 Stara Zagora, Bulgaria; nadiab@abv.bg; 3 Department of Physiology, Pathophysiology and Pharmacology, Medical Faculty, Trakia University, 6000 Stara Zagora, Bulgaria; wboneva@yahoo.co.uk; 4 Department of Social Medicine, Health Management and Disaster Medicine, Faculty of Medicine, Trakia University, 6000 Stara Zagora, Bulgaria; veselin.ivanov@trakia-uni.bg

**Keywords:** biopolymers, *Silybum marianum*, mechanical properties, antioxidant activity

## Abstract

Silymarin—a standardized extract from the seeds of milk thistle (*Silybum marianum* L. Gaertn.)—is mainly used for the treatment of hepatitis and other liver diseases. In recent years, the attention of researchers has been directed to its use in dermatology and wound treatment. Despite the promising results, there are still many unresolved issues in this area. The aim of the present study is to develop and characterize hydrogel chitosan–pectin films containing silymarin as an active ingredient with potential medical application. Six variants of hydrogel films (control and silymarin-loaded) were obtained from chitosan and pectin solutions by the casting method and analyzed in terms of their physicochemical, structural, mechanical and optical properties, as well as the in vitro dissolution profile of silymarin. The highest tensile strength was measured for the chitosan-based films—23.35 ± 1.74 MPa (control) and 22.01 ± 2.67 MPa (silymarin-loaded), while the barrier properties to UV and visible light were the strongest for chitosan–pectin films with silymarin. The antioxidant potential of the films was determined by DPPH assay and it was found that the variants with silymarin have over 20 times higher antioxidant activity (from 2.020 ± 0.048 to 2.106 ± 0.190 mg TE/g) than the corresponding controls. The results showed that chitosan–pectin films with incorporated silymarin could find application as potential hydrogel dressings in the therapy of wounds and superficial burns.

## 1. Introduction

Milk thistle (*Silybum marianum* L. Gaertn.) is an annual or biennial herbaceous plant native to North Africa and Southern Europe, but it is widely cultivated throughout Eurasia, North and South America as well as southern Australia due to its medicinal and antioxidant properties [1,2]. The seeds of *S. marianum* are rich in polyphenolic compounds belonging to the flavonolignan group. The standardized extract obtained from the seeds is known as silymarin (SM), which contains 65–80% flavonolignans—silybin, silydianin, silychristin, and isosilybin, with silybin being the predominant component [2,3].

For decades, silymarin has been primarily used in the treatment of hepatitis, alcoholic liver injury, cirrhosis, and other hepatic disorders [4,5]. More recently, research attention has turned to its potential applications in dermatology and wound healing. Topical application of silymarin has shown protective effects against chemical, radiation-induced, and UVB-induced skin damage [5,6]. A pluronic–lecithin organogel containing silymarin has been developed, demonstrating beneficial effects in patients with atopic dermatitis. Topical application of the silymarin-loaded organogel significantly alleviated inflammatory symptoms such as erythema, edema, and inflammation [7].

Reports on the topical application of silymarin-based formulations for wound healing remain limited. Animal studies have demonstrated beneficial effects in various wound models, with silymarin administered in the form of an ointment [8], dissolved in polyethylene glycol [9], or as a cream [10]. Other researchers have applied solutions of silybin (silibinin) and observed accelerated wound healing, along with increased levels of proline, collagen, N-acetylgalactosamine, and N-acetylglucosamine [11]. Aslan et al. investigated the molecular mechanisms of silymarin’s topical effects on wounds and reported re-epithelializing and anti-inflammatory actions, suggesting its potential as a therapeutic agent for wound treatment [4]. Positive outcomes have also been reported in human burn wound therapy when silymarin was administered systemically [12].

Despite these promising findings, many challenges remain regarding the topical use of silymarin in wound healing. Its poor solubility in both water and oils [13] presents technological difficulties in developing formulations that ensure homogeneous drug distribution and adequate bioavailability at the wound site. Furthermore, traditional topical dosage forms (ointment, cream, or gel) are not always optimal for wound application.

Polymeric wound dressings, particularly hydrogel-based, offer several advantages such as exudate absorption, maintenance of a controlled moist environment in the wound-conducive to healing and epithelialization, and the ability to act as carriers for various active substances [14,15]. Hydrogels for biomedical applications can be prepared from either natural or synthetic polymers that meet the requirements for biocompatibility, non-toxicity, mechanical strength, and biodegradability [16]. However, biopolymers derived from natural sources, such as chitosan, sodium alginate, collagen, and sodium hyaluronate are advantageous due to their safety, intrinsic biological activity, and environmental friendliness.

Over the past two decades, chitosan has emerged as one of the most widely used materials in biomedical applications, including wound care [17]. Chitosan is a cationic polysaccharide obtained through N-deacetylation of chitin, a major component of the exoskeletons of arthropods, most fungi, and certain algae. Like chitin, chitosan is biocompatible, non-toxic, and biodegradable, and it also exhibits mild fungistatic and bacteriostatic activity.

Various chitosan-based biomaterials have been developed through physicochemical or chemical modifications [17]. For example, Feng et al. developed a chitosan hydrogel film modified with an aptamer, capable of binding and releasing multiple therapeutic proteins in a controlled manner [18]. Another chemical modification approach involves crosslinking N-succinyl chitosan hydrogels with an aldehyde-derivatized dextran polymer. Such hydrogels have been reported to promote healing and prevent adhesions between adjacent damaged tissues when applied to surgical or traumatic wounds [19].

Recent studies have also focused on physically crosslinked chitosan hydrogels. The polycationic nature of chitosan enables its interaction with natural or synthetic polyanions. Pectins, which are anionic polysaccharides extracted from plant materials (commonly fruits and vegetables), can form thermoreversible gels with chitosan solutions. These hydrogels can effectively prolong the release of hydrophobic drugs [20]. The formation of physically crosslinked hydrogels is attributed to electrostatic attraction between the amine groups of chitosan and the carboxyl groups of pectin, along with extensive hydrogen bonding between the two polysaccharides, resulting in the formation of a polyelectrolyte complex. The structure of such complexes has been confirmed using FT-IR spectroscopy [21].

To the best of our knowledge, no studies have yet been reported on the development of hydrogel films based on chitosan and pectin incorporating silymarin as the active component. The objective of the present study is to formulate and characterize mixed chitosan–pectin active films containing silymarin, with potential application as hydrogel wound dressings.

## 2. Results and Discussion

**Figure 1:**
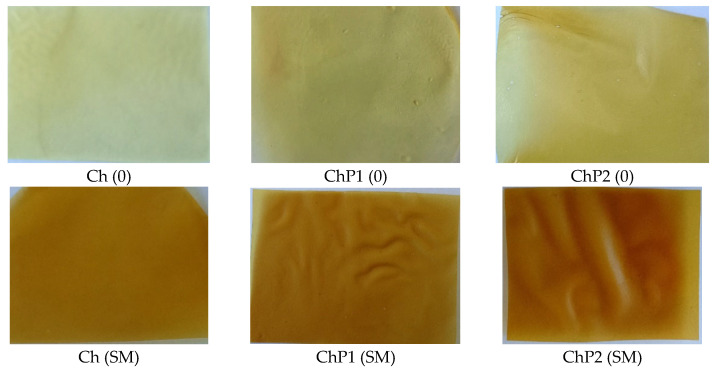
Photographs of the obtained chitosan and chitosan–pectin hydrogel films: control (Ch (0); ChP1 (0); ChP2 (0)) and silymarin-loaded (Ch (SM); ChP1 (SM); ChP2 (SM)).

The hydrogel films were prepared by solvent casting method with subsequent drying at controlled temperature until complete removal of the solvent. The correct selection of a suitable solvent and method for dissolving and mixing the biopolymers and other functional and active ingredients is essential for obtaining a homogeneous film-forming mixture (FFM) and, accordingly, a homogeneous flexible film. Chitosan dissolves at low pH and for this reason, 1% aqueous acetic acid solution was used as a solvent. To avoid particle sticking, trapping of air bubbles and aggregate formation, the chitosan powder was first mixed with small amounts (up to 15%) of ethanol and water, and then the diluted acetic acid was added. Silymarin is only sparingly soluble in water but readily soluble in 95% ethanol, which was therefore used as a solubilizing medium. After its incorporation into the chitosan or the mixed chitosan–pectin solutions, a stable colloidal system was formed, and the resulting films were homogeneous, without brittle areas or air bubbles. In the control samples, the silymarin solution was replaced with an equivalent volume of ethanol. The preparation of the composite films was carried out according to the above-described dissolution procedure with preliminary dry mixing of chitosan and pectin. Photographs of the resulting hydrogel film variants are presented in Figure 1.

### 2.1. Physicochemical Analysis of Hydrogel Chitosan and Chitosan–Pectin Films

The results of the physicochemical analyses of the films with and without silymarin are presented in Table 1.

The thickness of the films ranged from 0.078 to 0.121 mm, depending on their composition. Generally speaking, the thickness of the obtained films depends on the concentration of the biopolymer, the technology used and the drying conditions. The most popular method for obtaining films with controlled thickness is the use of a constant amount of film-forming mixture for a certain area. In the present study, the same ratio was applied for the different film variants—0.580 g FFM/cm^2^. The difference in thickness between chitosan and mixed chitosan–pectin films is mainly due to the interaction between chitosan and pectin. On the other hand, a significant increase in thickness is observed in the chitosan–pectin films with silymarin included. This change is due to the increased solids content in FFM and possible interactions between the components of silymarin and the molecules of the biopolymers.

In all experimental variants (control and silymarin-loaded), the pH of the 10% aqueous film extract was between 5.12 and 4.75. A slight decrease in pH was observed with increasing pectin content in the hydrogel matrix.

The residual moisture content for all formulations was between 10.67% and 14.57%. Water activity was low (0.321–0.362). It is well established that when water activity (aw) is below 0.600, microbial growth in the product is inhibited and hydrolytic processes are halted [22,23]. The obtained values therefore suggest good stability during prolonged storage at room temperature.

### 2.2. Fourier Transform Infrared Spectroscopy (FTIR)

**Figure 2:**
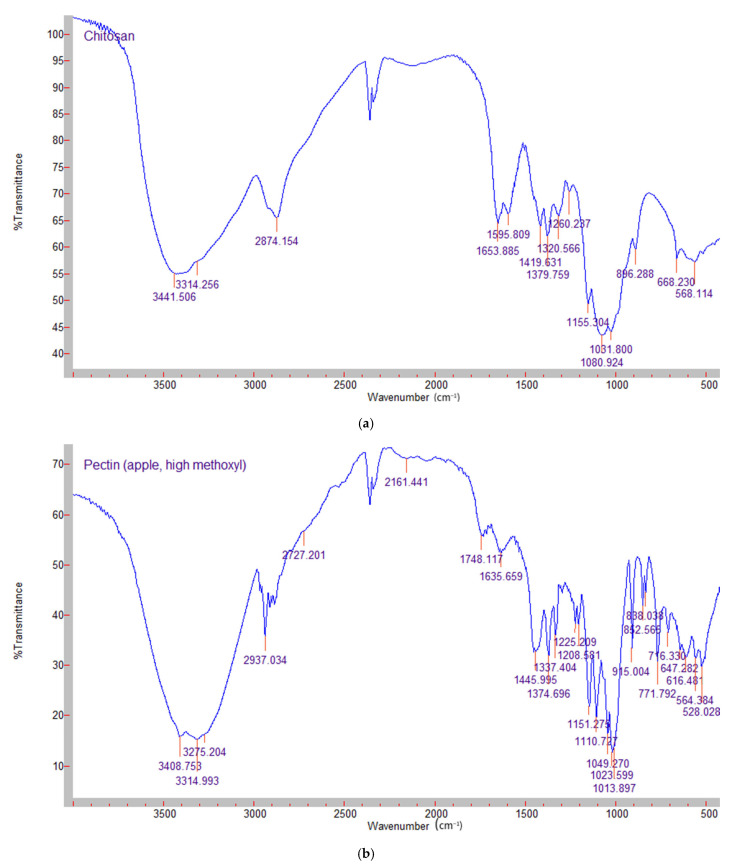

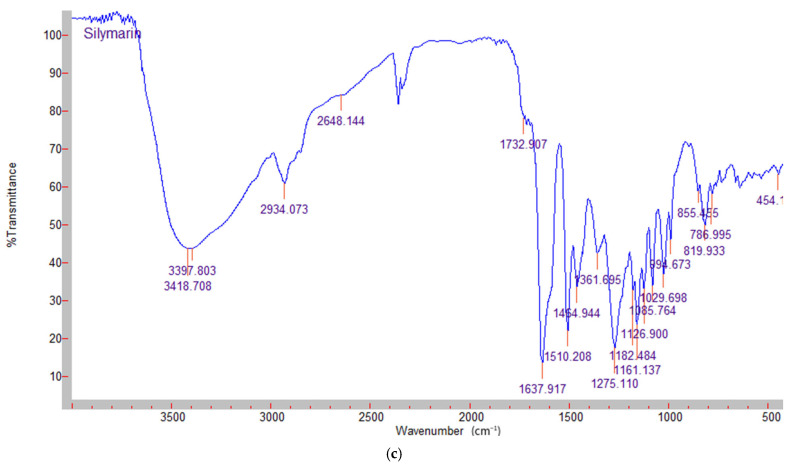
FTIR spectra of (**a**) chitosan; (**b**) high metoxyl apple pectin; (**c**) silymarin.

**Figure 3:**
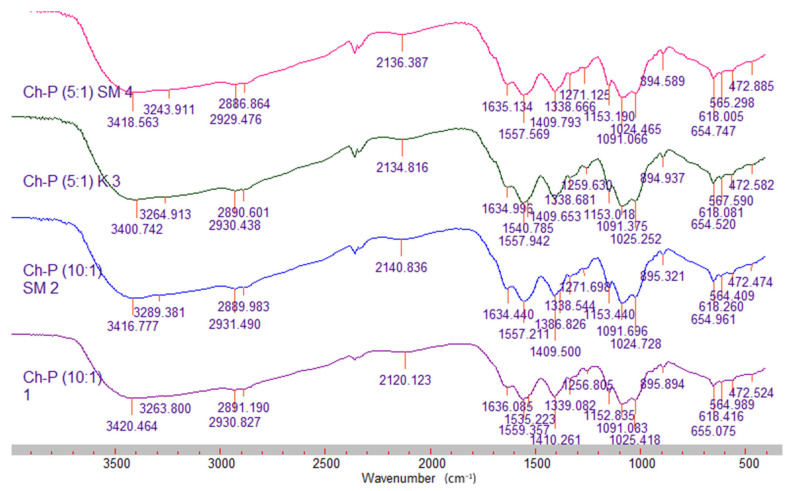
FTIR spectra of the mixed films–control and silymarin-loaded.

The FTIR spectra of chitosan, apple pectin and silymarin are shown in Figure 2 and Figure 3 presents the spectra of the mixed films–control and silymarin-loaded. In the chitosan powder spectrum, a strong band at around 3314 cm^−1^ was observed, corresponding to N-H and O-H stretching and the intramolecular hydrogen bonds. The absorption band around 2874 cm^−1^ can be assigned to C-H stretching. These bands are characteristics features of polysaccharides and were found in other polysaccharide spectra as well [24]. The presence of residual N-acetyl groups is confirmed by the bands at around 1653 cm^−1^ (C=O stretching of amide I) and 1320 cm^−1^ (C-N stretching of amide III), respectively.

The FTIR spectrum of pectin is shown in Figure 2b. As with chitosan, a broad absorp-tion band around 3314 cm^−1^ was observed, corresponding to OH-stretching due to inter- and intramolecular hydroxyl groups. The peak at 2937 cm^−1^ was related to the vibrations of C-H bonds. The absorption peaks at 1745 and 1635 cm^−1^ correspond to the esterified and free carboxyl groups of galacturonic acid. The peaks in the region of 1350–1450 cm^−1^ were reported to be related to esterified CH_3_ groups [25]. The silymarin spectrum (Figure 2c) shows the vibration peaks at 1638 cm^−1^, 1084 cm^−1^ (benzopyran ring) and 820 cm^−1^ (aromatic ring). The characteristic absorption pattern of silymarin (“fingerprint region”) is between 600 cm^−1^ and 1400 cm^−1^ [26].

The FTIR spectra of the mixed chitosan–pectin films showed shifts and decreases in the intensity of the characteristic peaks for both polymers, especially in the region of 700–1700 cm^−1^. The observed changes in absorption are most likely due to interactions between the charged groups (–NH^3+^ and –COO−) of the two polymers, which lead to changes in their initial vibrations [27]. When comparing the spectra of the control films with the silymarin-loaded ones, an increase in intensity and a shift in the peaks at 1257 cm^−1^, 1557 cm^−1^, 2141 cm^−1^ and 3420 cm^−1^ were observed in the later, which is indicative of an interaction between silymarin and the biopolymer components.

### 2.3. Mechanical Properties

Mechanical properties are among the most important parameters for assessing film quality, regardless of whether the films are intended for packaging applications or for use as hydrogel wound dressings. These properties also provide insight into the durability and stability of such materials during processing, transportation, and storage [27]. Tensile strength (TS) is defined as the ability of a film to resist tearing under tensile stress and reflects the maintenance of structural integrity. Elongation at break (EAB) represents the maximum extensibility of the film under tensile stress before breaking. Young’s modulus, also known as the elastic modulus, predicts the tendency of a film to stretch under tension or shorten under compression. Ultimately, the mechanical behavior of the films depends on the physicochemical properties of the polymers used, the added plasticizers and other components, intermolecular interactions, water content, and microstructure [28].

As shown in Table 2, the mechanical properties of the films varied considerably depending on the biopolymer composition and on the presence or absence of the active compound, silymarin. Regarding TS, the highest values were recorded for the chitosan films, specifically 23.35 MPa and 22.01 MPa. These results are consistent with those reported by Qin et al. [29] and Pereda et al. [30] for pure chitosan films, and significantly higher than those obtained by Ojagh et al. [31]. Conversely, ref. [28] reports higher TS values for control chitosan films (42.91 MPa). Such differences are most likely attributable to variations in the chitosan used and the film preparation conditions. Incorporation of silymarin into the chitosan film Ch (SM) did not significantly affect TS but markedly increased EAB values, which rose from 25.03% to 90.15%. Similar EAB values for control chitosan films were reported by [29], whereas [30] obtained slightly higher values. The elastic modulus (Young’s modulus) decreased in the sample containing silymarin.

Analysis of the control chitosan–pectin films showed a substantial reduction in TS compared with the chitosan film, while EAB values were only marginally affected. The lowest TS (14.57 MPa) was observed in ChP2 (0), where the pectin content was the highest (20% relative to chitosan mass). In a study by Younis and Zhao [27] on edible pectin–chitosan films, the highest TS value (6.49 MPa) was found in the formulation with the greatest pectin content. Unlike the present work, their films contained pectin as the predominant component and were produced using a different fabrication technique, which may explain the discrepancies in the results.

The mechanical properties of the mixed films containing silymarin differed notably from those of their respective controls. A decrease in TS and Young’s modulus was observed, accompanied by an increase in EAB. The explanation for this phenomenon can be sought in the plasticizing effect of silymarin on the biopolymer matrix. Plasticizers are usually low molecular weight compounds with many OH-groups, compatible with the respective biopolymer, which, added in optimal concentrations, improve the elastic and plastic properties of polymers. The plasticizer molecules penetrate between the polymer macromolecules, which changes the intermolecular interactions and increases the free volume and mobility of the chains. In the present study, glycerol was used as a plasticizer in the same concentration for all FFMs. The strong increase in EAB values and corresponding decrease in TS in films with silymarin is indicative of the influence of silymarin on the film structure. The changes in mechanical properties indicate that the film structure is altered compared to controls and is characterized by a greater tendency to deformation and increased flexibility. The change in the film structure is most likely due to intermolecular interactions between the components of silymarin and chitosan molecules. These interactions lead to a reduction in the bonds that exist between the biopolymer chains, which causes disruptions in the film network and consequently changes in the mechanical response. A similar trend in chitosan films with incorporated polyphenolic plant extracts has been observed by other authors. Shahbazi reported lower TS values in chitosan films loaded with plant extracts compared with the corresponding control film. The study used grape seed extract and observed an approximately 20% reduction in TS [28]. In a comparative study by Bajić et al. on chitosan films loaded with extracts from oak, hops, and algae [32], it was found that all three extracts caused a significant decrease in TS and an increase in EAB.

Nevertheless, it should be noted that in all mixed-film variants, TS remained above 11 MPa, indicating good film strength.

### 2.4. Optical Properties

#### 2.4.1. Color

The color parameters of the films are presented in Table 3. The ΔE value was calculated as the total color difference between each control film and its corresponding silymarin-loaded film.

Among the control samples, the pure chitosan film Ch (0) exhibited the highest L (lightness) value. As the amount of pectin increased, L decreased, reaching 66.64 in ChP2 (0). A further decrease in L was observed in the corresponding silymarin-containing films. Regarding the a (redness) and b (yellowness) parameters, the lowest values were recorded for the chitosan film. In the mixed chitosan–pectin films, the a and b values increased to 13.48 and 43.55, respectively (ChP2 (0)), indicating a predominance of the yellow color component. The change in the color parameters in the mixed films is due to the pectin component. Unlike citrus pectin, which is white in color, the color of apple pectin varies from beige to light brown. Its aqueous solutions also have a similar color. For this reason, darker films with higher positive values for a and b were obtained from FFMs containing pectin with a predominance of the yellow component.

Silymarin extract is yellow in color. Its ethanol solutions also have a saturated yellow color. In the films, containing silymarin, the a and b parameters increased significantly and reached 23.49 ± 1.34 and 63.30 ± 1.40, respectively. The high ΔE values reflect the pronounced color differences between the control films and the silymarin-loaded films, which also corresponds to the observed visual appearance.

#### 2.4.2. Light Transmission

**Figure 4:**
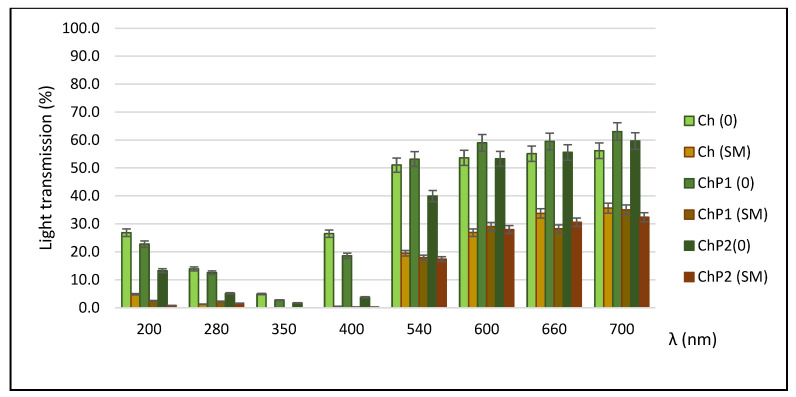
Light transmission of the developed films.

The light transmission of the films in the UV–VIS region is shown in Figure 4.

Barrier properties against electromagnetic radiation, particularly in the UV range, are an important parameter in the development of biopolymer films, whether they are intended for food packaging or for hydrogel wound dressings. The ability of a packaging material to act as a barrier to electromagnetic energy enhances photostability and prevents photooxidation of the packaged product. In wound applications, uncontrolled exposure to UV light may increase inflammation, oxidative stress, and hyperpigmentation, potentially hindering the healing process. An assessment of the films’ light transmission at specific wavelengths (200–700 nm) showed that the silymarin-loaded variants almost completely absorbed UV radiation in the 200–400 nm region. Like many other polyphenolic plant extracts, silymarin exhibits strong absorption in the ultraviolet (UVA/UVB) range. The main absorption peak of silymarin is at 286 nm with an additional one at 320 nm. After incorporation into chitosan and mixed chitosan–pectin matrices, their transmittance in the UV range (≤400 nm) is almost completely blocked, indicating a photoprotective effect against UV damage. In the visible range, these films also exhibited superior barrier properties compared to their respective controls.

### 2.5. Antioxidant Properties

The antioxidant properties of the developed films were evaluated by determining their inhibitory activity toward DPPH. The results are expressed as Trolox equivalents (Table 4).

As can be seen from the data in Table 4, the control samples have weak antioxidant activity due to the presence of chitosan and pectin [33,34]. However, the films with silymarin exhibit over 20 times higher antioxidant activity. The antioxidant activity of silymarin has been proven by many in vivo and in vitro studies [35]. Pientaweeratch et al. have studied the inhibitory effect of silymarin towards DPPH and ABTS and have found promising antioxidant potential [36]. In another in vitro study, free radical scavenging activity of silymarin was shown by four different assays [37]. Our results confirmed that after inclusion in the developed hydrogel films, silymarin retained its antioxidant activity.

### 2.6. Dissolution Study of Silymarin

**Figure 5:**
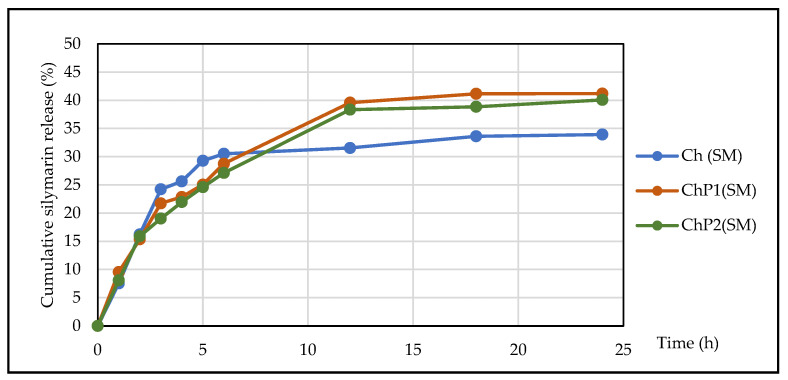
Silymarin dissolution profiles from silymarin-loaded hydrogel films.

Figure 5 shows the in vitro dissolution profile of silymarin in a 150 mL phosphate-buffered saline (PBS).

The results showed that the developed films provide prolonged release of silymarin. The release rate was the highest during the first 5 h, after which it decreased gradually and reached equilibrium after the 12th hour. The data shown in Figure 4 also confirm that the rate and extent of silymarin release depends on the composition of the hydrogel carrier. The lowest values were obtained for the chitosan film, while for the samples with chitosan–pectin films, the extent of silymarin release reached 41.20% of the applied amount.

## 3. Materials and Methods

### 3.1. Materials

Silymarin was supplied by Sopharma AD (Sofia, Bulgaria). Chitosan (degree of deacetylation 85%, Mw 310,000–375,000) was obtained from Biosynth (Staad, Switzerland). High-methoxyl apple pectin (DE 68%) was purchased from Herbstreith & Fox (Neuenbürg, Germany). Acetic acid, methanol, ethanol, glycerol, DPPH (2,2-diphenyl-1-picryl-hydrazyl), and Trolox (6-hydroxy-2,5,7,8-tetramethylchromane-2-carboxylic acid) were supplied by Sigma-Aldrich (Taufkirchen, Germany). All chemicals and reagents used were of analytical grade and were employed without further purification.

### 3.2. Preparation of Hydrogel Films (With and Without Silymarin)

The compositional formulations of the six developed hydrogel film-forming mixtures (FFM) are presented in Table 5. Each formulation was prepared to yield a final volume of 100 mL of FFM.

The preparation process included an initial stage of dry mixing of chitosan and pectin, followed by the gradual addition, under constant stirring, of an ethanolic solution of silymarin (20 mg/mL), distilled water, and a 1% *v*/*v* acetic acid solution. In the control samples, the silymarin solution was replaced with an equivalent volume of ethanol.

The resulting mixtures were homogenized for 30 min at 800 rpm, then treated with ultrasound (VEVOR TH-30A, Shanghai, China) to remove air bubbles. The obtained hydrogel films were formed by the casting method followed by drying. Initially, the film-forming mixtures (FFM) were poured into polytetrafluoroethylene (PTFE) non-stick trays at a ratio of 0.580 g FFM/cm^2^ and subsequently dried at 50 °C (20 kPa, SPT-200 Vacuum Drier, ZEAMiL, Krakow, Poland) for 16 h. The dried hydrogel films were detached and stored under controlled conditions (25 ± 2 °C; 50 ± 5% RH) until further analysis.

### 3.3. Infrared Spectroscopy Analysis (FTIR)

Dry silymarin, pectin, and chitosan, as well as the film variants with and without silymarin, were mixed with KBr. Each mixture was ground into a fine powder and pressed into pellets. FT-IR spectra were recorded using a Varian 660-IR FT-IR spectrometer (Agilent Technologies, Santa Clara, CA, USA) in the 4000–400 cm ^−1^ range with a resolution of 0.250 cm^−1^.

### 3.4. Physicochemical Analysis of Films

#### 3.4.1. Moisture Content

The moisture content (%) of the film samples was determined using a moisture analyzer, model DBS 60-3 (KERN, Balingen-Frommern, Germany).

#### 3.4.2. Water Activity

The water activity of the films was measured with a Novasina LabTouch CM-2 apparatus (Lachen, Switzerland).

#### 3.4.3. Thickness

The film thickness was measured with a digital micrometer Mitutoyo 293-832 (Kawasaki, Japan) with an accuracy of 0.001 mm ± 5%, at five randomly selected points of each sample.

#### 3.4.4. Water Absorption

The test was performed according to [38]. The film samples were cut into 3.0 × 3.0 cm pieces and weighed to determine their initial weight (m1). Each sample was immersed in 50 mL of distilled water for 1 h. After immersion, the samples were removed, drained for 1 min at a 45° angle to remove excess water, and reweighed (m2). Water absorption (A) was expressed as the increase in weight (g/g) and calculated using the equation:

(1) $$Waterabsorption=\frac{m2-m1}{m1},$$

#### 3.4.5. pH Measurement

The pH of 10% water extract of film samples was measured with Jenway 3000 pH meter.

### 3.5. Optical Properties

#### 3.5.1. Color Measurement

Instrumental color parameters of the control and silymarin-loaded films were determined using an NR200 Portable Digital Colorimeter (Huanyu, Wenzhou, China) based on the CIE Lab system. The *L*, *a*, and *b* values were measured against a white standard background (L* = 92.57, a* = 0.17, b* = −2.33). Each measurement was performed in five replicates. The total color difference (*ΔE*) was calculated according to the standard formula:

(2) $$∆E=\sqrt{{\left(L_{c}-L\right)}^{2}+{\left(a_{c}-a\right)}^{2}+{\left(b_{c}-b\right)}^{2}}$$

#### 3.5.2. Light Transmission (LT)

The light transmission (LT) of both control and silymarin-loaded films was measured using a UV–Vis spectrophotometer (Libra S22 UV–Vis, Biochrom, Holliston, MA, USA) at wavelengths of 280, 350, 400, 540, 600, 660, and 700 nm.

### 3.6. Mechanical Tests

Mechanical testing of the films was carried out according to the BDS EN ISO 527-3:2003 standard [39] using a Universal Mechanical and Tribological Tester, UMT (Bruker-CETR, Campbell, CA, USA), at the Open Laboratory on Experimental Micro and Nano Mechanics, Bulgarian Academy of Sciences. The tests were conducted at room temperature (20 ± 2 °C). Six specimens were tested for each sample, and the results are presented as mean values ± standard deviation.

### 3.7. Antioxidant Activity Measurement

The antioxidant activity of the films was determined using the DPPH radical scavenging assay, following the method of Brand-Williams et al. [40]. Film samples (0.2 g) were ground and extracted with 10 mL of 95% ethanol under magnetic stirring for 1 h, followed by centrifugation at 8000 rpm for 10 min at 4 °C (Beckman J2-21M; Beckman Coulter, Brea, CA, USA). The supernatant was collected and diluted with 80% methanol. A mixture containing 0.6 mL of DPPH methanolic solution (0.2 mM), 0.9 mL of methanol, and 0.5 mL of the supernatant was incubated for 60 min in the dark at room temperature. The absorbance was then measured at 517 nm. Antioxidant activity was calculated using a calibration curve prepared with Trolox (1.0–15.0 µg/mL) and expressed as Trolox equivalents (mg TE/g).

### 3.8. In Vitro Dissolution of Silymarin

Film samples (2 × 2 cm) were weighed and placed in 300 mL flasks containing 150 mL of phosphate-buffered saline (PBS, 0.1 M, pH 7.4). The flasks were kept in a shaking water bath (Memmert WTB, Schwabach, Germany) at 36.5 °C with constant agitation (70 rpm). At predetermined time intervals (1, 2, 3, 4, 5, 6, 12, 18, and 24 h), 2.0 mL aliquots were withdrawn, filtered through paper filters, and analyzed spectrophotometrically for silymarin content according to [7]. The absorbance of the filtrates was measured at 322 nm against PBS and compared with a calibration curve constructed from standard silymarin solutions in PBS (2–30 μg/mL, R^2^ = 0.999).

### 3.9. Statistical Analysis

Data were analyzed by Microsoft Excel 2016 software (Microsoft, Redmond, WA, USA) and expressed as the mean ± standard deviation (SD) of three replicates unless specified. Student’s *t*-test was used for comparisons by control and silymarin loaded films. The difference was statistically significant at *p* ≤ 0.05 level.

## 4. Conclusions

The present study examines the possibility for new pharmaceutical application of silymarin. Silymarin, a standardized extract from milk thistle, is traditionally used in the treatment of liver diseases but has shown promising experimental results when applied topically in wound therapy. The aim of the study was to develop and analyze hydrogel films based on chitosan and mixtures of chitosan and pectin, containing silymarin as an active ingredient. The evaluation of the obtained film variants was made based on their mechanical, physicochemical, optical and antioxidant properties, as well as on the kinetics of silymarin dissolution in a model system. It was found that films with silymarin had stronger barrier properties against UV and visible light and over 20 times higher antioxidant activity than the corresponding control films. The film CHP1 (SM) showed the best properties in all parameters. Therefore, it has a high potential to be used as a natural active hydrocolloid dressing for wounds and superficial burns. However, further studies are needed in relation to its potential medical application, including cytotoxic and microbiological analyses and evaluation of stability during sterilization and after long-term storage.

## Figures and Tables

**Table 1:** Physicochemical properties of chitosan and chitosan–pectin films, control and silymarin-loaded.

Formulation Code	Thickness (mm)	Moisture Content (%)	Water Activity (aw)	Water Absorption (g/g)	pH (10% Water Extract)
Ch (0)	0.094 ± 0.011	12.02 ± 0.77	0.321 ± 0.002	1.75 ± 0.11	5.12 ± 0.15
Ch (SM)	0.085 ± 0.010	10.67 ± 0.35	0.312 ± 0.004	2.13 ± 0.13	5.08 ± 0.13
ChP1 (0)	0.078 ± 0.007	13.71 ± 0.73	0.354 ± 0.005	1.66 ± 0.14	4.86 ± 0.20
ChP1 (SM)	0.118 ± 0.012	13.05 ± 0.11	0.341 ± 0.004	2.35 ± 0.12	4.85 ± 0.16
ChP2 (0)	0.088 ± 0.014	14.57 ± 0.81	0.362 ± 0.009	1.56 ± 0.11	4.76 ± 0.18
ChP2 (SM)	0.121 ± 0.008	13.83 ± 0.88	0.352 ± 0.011	2.51 ± 0.10	4.75 ± 0.14

Values represent means ± standard deviations (*n* = 3).

**Table 2:** Mechanical properties of chitosan and chitosan–pectin composite films, control and silymarin-loaded.

Formulation Code	Yield Strength (MPa)	Tensile Strength (MPa)	Young’s Modulus (MPa)	Elongation at Break (%)	Toughness (J/mm^3^)
Ch (0)	12.69 ± 1.21	23.35 ± 1.74	60.14 ± 3.02	25.03 ± 2.68	2.97 ± 0.26
Ch (SM)	8.35 ± 0.46	22.01 ± 2.67	39.17 ± 1.78	90.15 ± 11.13	6.15 ± 1.03
ChP1 (0)	13.30 ± 1.08	19.98 ± 1.20	182.09 ± 17.23	32.81 ± 6.62	3.52 ± 0.60
ChP1 (SM)	1.28 ± 0.32	12.72 ± 2.11	17.05 ± 1.25	127.35 ± 12.47	6.98 ± 1.09
ChP2 (0)	4.48 ± 0.33	14.57 ± 0.81	197.70 ± 18.40	22.34 ± 2.14	0.88 ± 0.19
ChP2 (SM)	3.55 ± 0.39	11.51 ± 2.27	84.40 ± 14.10	28.12 ± 4.17	1.30 ± 0.24

Values represent means ± standard deviations (*n* = 6).

**Table 3:** Color parameters of chitosan and chitosan–pectin composite films, control and silymarin-loaded.

Formulation Code	L	a	b	ΔE
Ch (0)	87.31 ± 1.88	1.01 ± 0.61	13.60 ± 4.02	-
Ch (SM)	61.73 ± 1.31	10.71 ± 0.53	37.84 ± 0.36	34.71 ± 7.36
ChP1(0)	81.29 ± 1.34	6.74 ± 1.64	31.79 ± 4.02	-
ChP1(SM)	54.72 ± 0.83	21.63 ± 0.65	44.32 ± 1.02	31.74 ± 1.56
ChP2(0)	66.64 ± 0.83	13.48 ± 0.91	43.55 ± 2.94	-
ChP2(SM)	53.26 ± 2.69	23.49 ± 1.34	63.30 ± 1.40	22.87± 4.85

Values represent means ± standard deviations (*n* = 5).

**Table 4:** Antioxidant activity of the developed films.

Control Films	Antioxidant Activity (mg TE/g)	Silymarin Loaded Films	Antioxidant Activity (mg TE/g)
Ch (0)	0.081 ± 0.012	Ch (SM)	2.060 ± 0.091
ChP1 (0)	0.114 ± 0.018	ChP1(SM)	2.106 ± 0.190
ChP2 (0)	0.093 ± 0.005	ChP2(SM)	2.020 ± 0.048

Values represent means ± standard deviations (*n* = 5).

**Table 5:** Composition of the different formulations for the preparation of 100 mL of film-forming mixtures (FFM).

Formulation Code	Chitosan (g)	Pectin (g)	Silymarin (g)	Ethanol 95% (mL)	1% Acetic Acid (mL)	Water (mL)	Glycerol (g)
Ch (0)	1.40	-	-	5.0	85.0	10.0	1.0
Ch (SM)	1.40	-	0.10	5.0	85.0	10.0	1.0
ChP1(0)	1.40	0.14	-	5.0	85.0	10.0	1.0
ChP1(SM)	1.40	0.14	0.10	5.0	85.0	10.0	1.0
ChP2(0)	1.40	0.28	-	5.0	85.0	10.0	1.0
ChP2(SM)	1.40	0.28	0.10	5.0	85.0	10.0	1.0

## Data Availability

The data presented in this study are available on request from the corresponding author.
